# Discovery of Potential Biomarkers for Postmenopausal Osteoporosis Based on Untargeted GC/LC-MS

**DOI:** 10.3389/fendo.2022.849076

**Published:** 2022-04-19

**Authors:** Jun Kou, Chunyang He, Lin Cui, Zhengping Zhang, Wei Wang, Li Tan, Da Liu, Wei Zheng, Wei Gu, Ning Xia

**Affiliations:** ^1^ College of Medicine, Southwest Jiaotong University, Chengdu, China; ^2^ Department of Orthopedics, General Hospital of Western Theater Command, Chengdu, China; ^3^ Department of Hyperbaric Oxygen, General Hospital of Western Theater Command, Chengdu, China; ^4^ Department of Spinal Surgery, Honghui Hospital, Xi’an Jiaotong University College of Medicine, Xi’an, China; ^5^ School of Automation, Chongqing University of Posts and Telecommunications Chongqing, Chongqing, China

**Keywords:** biomarkers, postmenopausal osteoporosis, metabolomics, mass spectrometry, gas chromatography, liquid chromatography

## Abstract

**Purpose:**

As an important public health problem, osteoporosis (OP) in China is also in an upward trend year by year. As a standard method for diagnosing OP, dual-energy X-ray absorptiometry (DXA) cannot analyze the pathological process but only see the results. It is difficult to evaluate the early diagnosis of OP. Our study was carried out through a serum metabolomic study of OP in Chinese postmenopausal women on untargeted gas chromatography (GC)/liquid chromatography (LC)–mass spectrometry (MS) to find possible diagnostic markers.

**Materials and Methods:**

50 Chinese postmenopausal women with osteoporosis and 50 age-matched women were selected as normal controls. We first used untargeted GC/LC-MS to analyze the serum of these participants and then combined it with a large number of multivariate statistical analyses to analyze the data. Finally, based on a multidimensional analysis of the metabolites, the most critical metabolites were considered to be biomarkers of OP in postmenopausal women. Further, biomarkers identified relevant metabolic pathways, followed by a map of metabolic pathways found in the database.

**Results:**

We found that there may be metabolic pathway disorders like glucose metabolism, lipid metabolism, and amino acid metabolism in postmenopausal women with OP. 18 differential metabolites are considered to be potential biomarkers of OP in postmenopausal women which are a major factor in metabolism and bone physiological function.

**Conclusion:**

These findings can be applied to clinical work through further validation studies. It also shows that metabonomic analysis has great potential in the application of early diagnosis and recurrence monitoring in postmenopausal OP women.

## Introduction

With over 200 million people worldwide with osteoporosis (OP) (1), the main features of the disease are low bone mineral density (BMD), bone loss, microstructure deterioration, and bone quality decline (2), which puts up the fracture vulnerability and the risk of individual hip, spine, and other bone fractures (3). From the clinical data, the prevalence and fracture rate of OP in postmenopausal women are much higher than those in elderly men, so OP is usually considered as a “woman’s disease” (4). OP has become a major health problem in developed countries. The existing data show that compared with other Caucasian populations, the prevalence of OP in the Chinese population is higher (5); this will inevitably lead to huge medical costs caused by osteoporosis in China.

As a standard method for diagnosing OP (6), dual-energy X-ray absorptiometry (DXA) cannot detect the pathological process of OP; these changes can only be displayed on DXA for many years. Some bone turnovers are now also used in the diagnosis and drug efficacy evaluation of patients with OP. Some of these markers have been clinically used to determine bone resorption, such as type I collagen cross-linked C-telopeptide (CTX), deoxypyridinoline, serum tartrate-resistant acid phosphatase 5b (TRACP5b), and type I collagen cross-linked N-telopeptide (NTX); there are also some indicators of bone formation, such as osteocalcin and procollagen type I N-terminal propeptide (P1NP) and bone alkaline phosphatase (BAP). In the case of the gold standard for the diagnosis and drug efficacy, no specific marker can be used to determine them. Therefore, it is difficult to evaluate the early diagnosis of OP.

More than 100 years ago, Sir Hans Krebs, an early biochemist, discovered the urea cycle and the citric acid cycle and made a pioneering study on metabolites for the first time. The metabonomics technology is widely used in clinical and biomedical research and has gradually become a new overall diagnostic tool using both advanced analytical technology and bioinformatics. Because it can reflect the current phenotype of specific biological systems, metabonomic measurement can really improve the understanding of pathophysiological process of disease progression and the discovery of new biomarkers for disease diagnostics or prognosis in various organisms (7). At present, there are many mass spectrometry (MS)-based high-throughput platforms, which can analyze 1,000–10,000 samples per day; it has been applied to a variety of metabolomics studies. Microfluidics and miniaturization of separation techniques, as a commonly used emerging technology, can analyze quickly and accurately (8).

Liquid chromatography (LC)–mass spectrometry and gas chromatography (GC)–mass spectrometry are the most common analytical platforms for mass spectrometry in metabolomics research. GC-MS is one of the most effective, repeatable, and commonly used analysis platforms in metabonomics research with the characteristics of robustness, excellent separation ability, selectivity, sensitivity, and reproducibility (9). Due to the ion suppression and matrix effect by co-eluting compounds, GC-MS obtains a higher chromatographic resolution than LC-MS to some certain extent (10). Because it can be used only to distinguish volatile compounds and low molecular weight (about 50–600 DA), chemical derivatization is required before GC-MS is used to detect polar, heat-resistant, and non-volatile metabolites, which makes GC-MS have an inherent limitation (11). LC-MS combines the separation ability of LC and the mass analysis ability of MS; it can not only separate pure or near-pure parts from a chemical mixture but also identify compounds with polymer specificity and detection sensitivity (12). Therefore, LC-MS often analyzes thermally labile, non-volatile, and polar compounds (13). According to the available literature, there are 6 metabonomics studies on patients with osteoporosis, of which two have studied the plasma of postmenopausal women with osteoporosis with nuclear magnetic resonance (NMR) (14) and LC-MS (15), respectively, and three have studied the serum of postmenopausal women with osteoporosis with NMR (16), GC-MS (17), and LC-MS (18) respectively; another study used LC-MS (19) to study the serum of patients with osteoporosis. They all used a single method; the recognition area of metabolites was relatively narrow, and the number of patients involved in most studies was small. Therefore, the results of these studies had some limitations. By combining the two technologies, we can make full use of the technical advantages of GC-MS and LC-MS to study metabolomics more comprehensively and accurately.

This study measured the metabolites in the serum of postmenopausal women with OP and postmenopausal women with normal BMD based on untargeted GC/LC-MS. Untargeted metabonomics can collect as much material information as possible and has a wide material coverage. Serum is easily available and contains molecules that represent the current state of the body and short-term changes. Compared with other compartments, it can better understand the metabolic processes of animal models and humans over a period of time (20). The serum of postmenopausal women with OP was collected in our hospital, and the serum of postmenopausal women with normal BMD without other basic diseases was selected for control. Then, by analyzing the differences of the two groups, biomarkers that can detect postmenopausal osteoporosis early were selected. This is the first metabolic study with our knowledge of OP in postmenopausal women in China based on untargeted GC/LC-MS.

## Material and Methods

### Participants

In this study, the case group comprised postmenopausal women diagnosed as OP during a physical examination in the General Hospital of Western Theater Command from June 2020 to June 2021. The inclusion criteria are as follows: (1) postmenopausal women with independent signing rights, (2) participants with OP who were definitely diagnosed with clinical manifestations combined with DXA, and whose T value of BMD of spine was less than -2.5. The exclusion criteria are as follows: (1) participants who suffer from a health complication that may impact bone metabolism, (2) participants who have received drugs or treatments that may affect BMD, and (3) participants with addiction aggression. In addition, the healthy control group was composed of age-matched postmenopausal volunteers who had normal BMD of spine and femur neck; the exclusion criteria for the control group were the same as in the case group. This study requires that all volunteers review and sign a form of informed consent carefully. At the same time, the Ethics Committee of the hospital (General Hospital of Western Theater Command) approved the clinical study.

### Sample Collection and Processing

Each serum sample was collected by volunteers on an empty stomach in the morning. First, the collected fresh whole blood was loaded into an untreated sterile non-anticoagulant tube at room temperature. After collection, it was allowed to stand for 30 min and centrifuged at 16,000 × g for 15 min at room temperature (20). Finally, the upper serum was collected in an Eppendorf tube, 300 μl of each serum was collected, in liquid nitrogen frozen for 30 s, and the stored temperature was -80°C for the next analysis.

We thawed the samples at room temperature. First, a 150-μl sample was added to a 1.5-ml Eppendorf tube, and 10 μl 3, 4-dichlorophenylalanine (0.3 mg/ml) with methanol dissolved in the tube was used as the internal standard, then the tube was vortexed for 10 s. Next, 450-µL mixtures of methanol and acetonitrile (2/1, vol/vol) were added and vortexed for 30 s, and the whole sample was extracted by ultrasonication in an ice water bath for 10 min and stored at -20°C for 30 min. The extract was centrifuged for 10 min (4°C 13,000 RPM). In a freeze concentration centrifugal dryer, the 150-µl supernatant was dried in a glass bottle. The glass-derived vial was filled with 80 μl of 15 mg/ml methoxyamine hydrochloride in pyridine. We first vigorously rotated the mixture for 2 min and then cultured it at 37°C for 90 min. 50 μl of BSTFA (with 1% TMCS) and 20 μl n-hexane were added, and the mixture was vigorously rotated for 2 min and derivatized for 60 min at 70°C. Finally, after the samples were left for 30 min at ambient temperature, they were analyzed by GC-MS.

A 150-μl sample was added into another 1.5-ml Eppendorf tube with an internal standard of 10 μl of L-2-chlorophenylalanine (0.3 mg/ml) dissolved in methanol (0.3 mg/ml); the test tube was rotated for 10 s. Next, a 450-μl mixture of acetonitrile and methanol (1/2, vol/vol) was added in ice-cold state and rotated for 1 min, and the whole sample was extracted for 10 min in an ice water bath using ultrasound and then left at -20°C for 30 min. The extract was centrifuged for 10 min (4°C 13,000 RPM). 0.22-μm microfilters were used to filter the 150-μl supernatant in the tube collected using a crystal syringe and then transferred to LC vials. The vials were left at -80°C and then were analyzed by LC-MS.

### Metabolite Measurement

The derivative was separated using an AHP-5MS fused-silica capillary column (30 m × 0.25 mm × 0.25 μm, Agilent J&W Scientific, Folsom, CA, USA); the derived samples were analyzed by GC-MS on an Agilent 7890B gas chromatography system and Agilent 5977B MSD system (Agilent Technologies Inc., CA, USA). A Vion IMS QTof Mass Spectrometer (Waters Corporation, Milford, USA) and ACQUITY UPLC I-Class system (Waters Corporation, Milford, USA) are used for LC-MS analysis of samples at the same time, and the metabolic spectra in ESI-positive ion and ESI-negative ion modes were obtained. For evaluating the data repeatability, QCs were injected every 10 samples throughout the analysis. Quality control samples (QC) are prepared by mixing the extracts of all samples in equal volume. The original data were processed by the Progenesis QI v2.3 (Nonlinear Dynamics, Newcastle, UK), and peak detection, peak identification, MS2Dec deconvolution, characterization, peak alignment, wave filtering, and missing value interpolation were performed. In each sample, all peak signal intensities were segmented and normalized according to the internal standards with a relative standard deviation (RSD) greater than 0.3 after screening. After the data were normalized, redundancy removal and peak merging were conducted to obtain the data matrix. For the extracted data, the ion peak with the missing value (0 value) >50% was deleted in the group, and the 0 value was replaced with half of the minimum value.

### Multivariate Data Analysis

To understand the metabolic variety of OP in postmenopausal Chinese women and postmenopausal Chinese women with normal BMD, principal component analysis (PCA), partial least square discriminant analysis (PLS-DA), and orthogonal projection to latent structure with discriminant analysis (OPLS-DA) are used as statistical analysis tools.

We performed these multivariate data analyses using the R Programming Language. PCA, an analytical pattern recognition tool without supervision, captures most of the variation of the whole data set with transforming high-dimensional data into a group of smaller orthogonal variables or components. PCA is usually applied to multiple data sets, and the generated two- or three-dimensional plots are visually compared to evaluate the differences (21). The spatial coordinates of each sample are composed of the projection score values on the plane composed of the first principal component and the second principal component, which can intuitively reflect the similarity or difference between samples. A unit variance scaling method was used for PLS-DA and OPLS-DA. PLS-DA is a method with supervision; by modeling the relationship of prediction space and response space, the potential corresponding variables to the principal components of principal component analysis are determined, and the covariance (PLS-DA score) between the two matrices is explained as much as possible, which can be used to predict the response of the population (22). Using MS data to perform OPLS-DA can more effectively facilitate the loading interpretation. By inversing the calculation of the coefficient, we obtained the model coefficient containing variable weights and drew it using color-coding coefficients to increase the interpretability of the model (23). To assess the PLS-DA and OPLS-DA, two parameters, R2Y and Q2, are used. R2Y shows the possibility of a difference between the square sums of all Xs and Ys. Q2 can show the percentage of cumulative cross validation in total predictable changes in current potential variables. The higher R2Y coefficient values and Q2 coefficient values (>0.5) show better ability of discrimination and prediction (24). At the same time, the PLS-DA model is cross validated by a 200-times permutation test; the permutation test is evaluated by cross validation, and the correlation coefficients R2 and Q2 of cross validation are used to verify whether there is overfitting. If the Q2 regression line intercept on the Y-axis is less than 0, the model can be reliable and effective, which is not overfitting (25).

### Find Key Biomarkers and Analysis Metabolic Pathway

After multidimensional statistical analysis, we screened out the metabolites with an absolute value of p < 0.05 and variable importance for the projection (VIP) >1.0, which is considered to have great potential as a potential biomarker of OP in postmenopausal women (26). Then, the Kyoto Encyclopedia of Genes and Genomes (KEGG) was searched to find metabolic pathways related to these key metabolites, the relevant literature was reviewed to verify their pathological relationship with OP, and the screened metabolic pathway was finally drawn. At the same time, we will also analyze the correlation between the metabolites we screened and the other two bone turnover markers (TRACP5b and BAP).

### Statistical Methods

The means ± SDs were expressed. The Kolmogorov–Smirnov test was used to inspect the normality and homogeneity of variance of all the data. A comparative study of the results from 2 groups was conducted by Student’s 2-sided t-test, and a 1-way analysis of variance was performed to explain differences in more than 2 groups. The correlation between two continuous variables was assessed using Pearson correlation analysis. The significance standard is p < 0.05. The Statistical Package for the Social Sciences, version 25.0 (SPSS, Chicago, IL), is used to statistical analysis.

## Results

### Participants

During this study, 50 postmenopausal osteoporosis women and 50 healthy postmenopausal women whose BMD was normal were eventually incorporated in our study. As shown in the **Table 1**, the healthy control group basically matched to those in the case group from the age, menopausal age, body mass index, and T value of the spine BMD. Through statistical data analysis, the values of these conform to the normal distribution.

**Table 1:** Participant characteristics at the time of sampling.

Characteristics	Case group	HCG	p*^a^*
Number of participants	50	50	—
Age (y, mean ± SD)	69.3 ± 9.3	66.3 ± 10.0	0.130
Menopausal age (y, mean ± SD)	49.5 ± 5.4	48.9 ± 5.6	0.562
BMI (kg/m^2^, mean ± SD)	23.8 ± 3.2	23.5 ± 4.4	0.672
BMD of spine (T, mean ± SD)	-3.2 ± 0.3	0.05 ± 0.6	—

^a^Calculated by Student’s t-tests for continuous variables and chi^2^ tests for categorical variables between case group and healthy control group.

BMI, body mass index; SD, standard deviation; HCG, healthy control group; BMD, bone mineral density.

### Untargeted GC/LC-MS Analysis of Samples

We performed a comprehensive metabolomic analysis of the serum of two groups of postmenopausal women. The identification of compounds is based on the accurate mass number, secondary fragments, and isotopic distribution, and the Human Metabolome Database (HMDB), LIPID MAPS (v2.3), and A Metabolite Mass Spectral Database (METLIN) are used for qualitative analysis. 48 compounds by GC-MS and 306 compounds by LC-MS were identified respectively in serum, including fatty acids, amino acids, and some carbohydrates. After multivariate analysis, according to the value of VIP, fold change (FC), and P of metabolites, 18 metabolites are considered as potential biomarkers of postmenopausal women with OP (**Table 2**). **Table 2** shows the specific metabolites designated by GC/LC-MS.

**Table 2:** Summary of potential biomarkers of the case group by serum GC/LC-MS analysis.

Metabolite	Status*^a^*	VIP value*^b^*	FC*^c^*	p*^c^*	Data origin	Pearson correlations	
						TRACP5b	BAP
Isothreonic acid	↑	1.91	2.1	<0.001	GC-MS	0.18	0.10
Ornithine	↑	1.07	1.5	<0.001	GC-MS		
Lactobionic acid	↑	2.36	2.8	<0.001	GC-MS		
Tartaric acid	↓	1.29	0.6	<0.001	GC-MS		
Glyceric acid	↓	1.29	0.7	<0.001	GC-MS		
Stearic acid	↓	1.10	0.8	<0.001	GC-MS		
PC	↑	1.60	3.6	<0.001	LC-MS	0.12	-0.006
Linoleic acid	↓	6.50	0.005	<0.001	LC-MS		
LysoPC	↑	4.05	26.2	<0.001	LC-MS		
PE	↑	3.74	19.7	<0.001	LC-MS		
DG	↓	3.62	0.004	<0.001	LC-MS		
PS	↓	2.02	0.07	<0.001	LC-MS		
SM	↓	2.00	0.06	<0.001	LC-MS		
Docosahexaenoic acid	↓	1.96	0.04	<0.001	LC-MS		
D-Glucose	↓	1.32	0.2	<0.001	LC-MS		
Lipoxin C4	↑	1.29	11.1	<0.001	LC-MS		
Heneicosanedioic acid	↓	1.16	0.005	<0.001	LC-MS		
PA	↓	1.15	0.03	<0.001	LC-MS		

^a^Relative concentrations compared to healthy controls: ↑ = upregulated, ↓ = downregulated.

^b^Correlation coefficient and VIP value were obtained from OPLS-DA analysis.

^c^Fold change between PWOP patients and healthy controls.

^c^p value determined from Student’s t-test.

PC, phosphatidylcholine; LysoPC, lysophosphatidylcholine; PE, phosphatidylethanolamine; DG, diacylglycerol; PS, phosphatidylserine; SM, sphingomyelin; PA, phosphatidic acid; FC, fold change; HC, healthy control; VIP, variable importance for projection.

### Multivariate Data Analysis Base on MS Data

**Figure 1:**
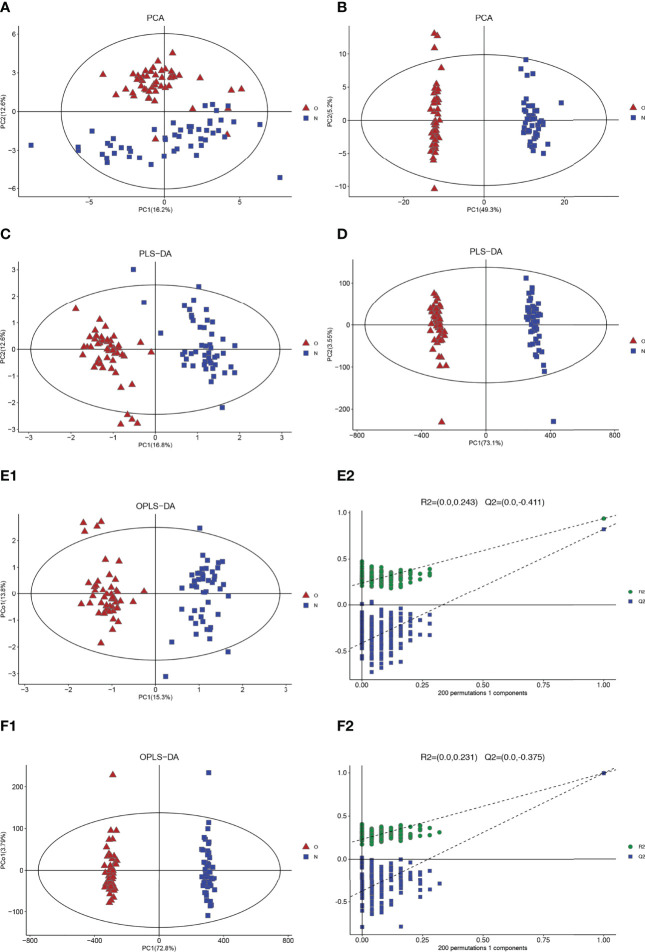
Multivariate date analysis of date from serum between the case group (O red triangle) and healthy control group (N blue squares) base on GC/LC-MS. **(A)** PCA score plots based on the GC-MS. **(B)** PCA score plots based on the LC-MS. **(C)** PLS-DA score plots. **(D)** PLS-DA score plots. **(E1, 2)** OPLS-DA score plots (left panel) and statistical validation of the corresponding OPLS-DA model by permutation analysis (right panel) based on the GC-MS. **(F1, 2)** OPLS-DA score plots (left panel) and statistical validation of the corresponding OPLS-DA model by permutation analysis (right panel) based on the LC-MS. The two coordinate points are relatively far away on the score map, indicating that there is a significant difference between the two samples, and vice versa. The elliptical region represents a 95% confidence interval.

Through the PCA score plots (**Figures 1A, B**), it can be seen that there is a significant difference in serum samples between the postmenopausal women with the OP group and the healthy control group, which indicated that the OP group and the control group have a significant and complete difference.

Two clusters corresponding between the case group and control group can be highlighted by the two detection methods from the PLS-DA plots. R2Y and Q2 are 0.940 and 0.813 (GC-MS), 0.966, and 0.994 (LC-MS), respectively, in PLS-DA (**Figures 1C, D**). These results show that the model has good recognition and prediction ability. R2Y and Q2 were 0.940 and 0.824 (GC-MS) and 0.996 and 0.995 (LC-MS) in OPLS-DA, respectively (**Figures 1E, F**), which also reveals that the model with good discrimination is predictive to be accurate and accurately defined.

**Figure 2:**
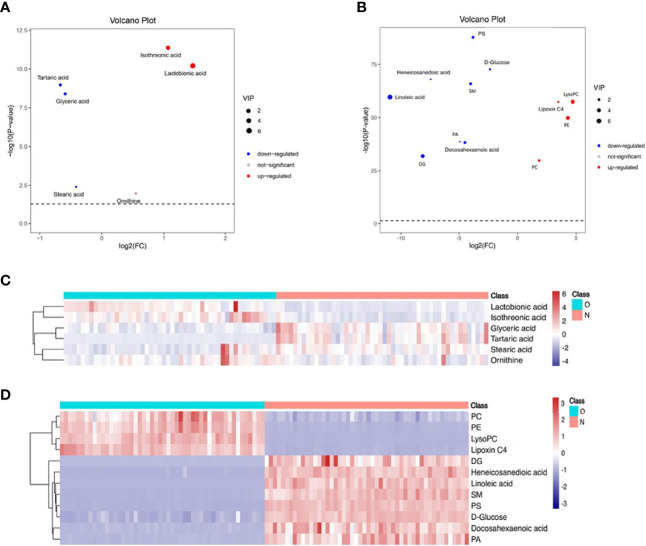
Volcano plot and hierarchical clustering based on the GC/LC-MS of serum metabolites obtained from the case group (O blue) and healthy control group (N red). **(A)** Volcano plot based on GC-MS. **(B)** Volcano plot based on LC-MS. **(C)** Hierarchical clustering based on GC-MS. **(D)** Hierarchical Clustering based on LC-MS. In **(A, B)**, the blue dot represents metabolite with a downward trend, red represents metabolites with an upward trend, and the gray origin represents that the change of metabolites is not obvious. The area size of the point is related to the VIP value. In **(C, D)**, the color from blue to red illustrates that metabolites’ expression abundance is low to high in hierarchical clustering. PC, phosphatidylcholine; LysoPC, lysophosphatidylcholine; PE, phosphatidylethanolamine; DG diacylglycerol; PS, phosphatidylserine; SM, sphingomyelin; PA, phosphatidic acid.

The volcanic map shows the p value and fold change value, thus proving the effectiveness of differential metabolites. Hierarchical clustering is carried out through the expression of all metabolites with significant differences, which can reflect the relationship among samples and the metabolite expression differences among different samples more directly. **Figure 2** indicates that the differences of the metabolite we chose are significant.

### Potential Biomarkers and Pathway Analysis

**Figure 3:**
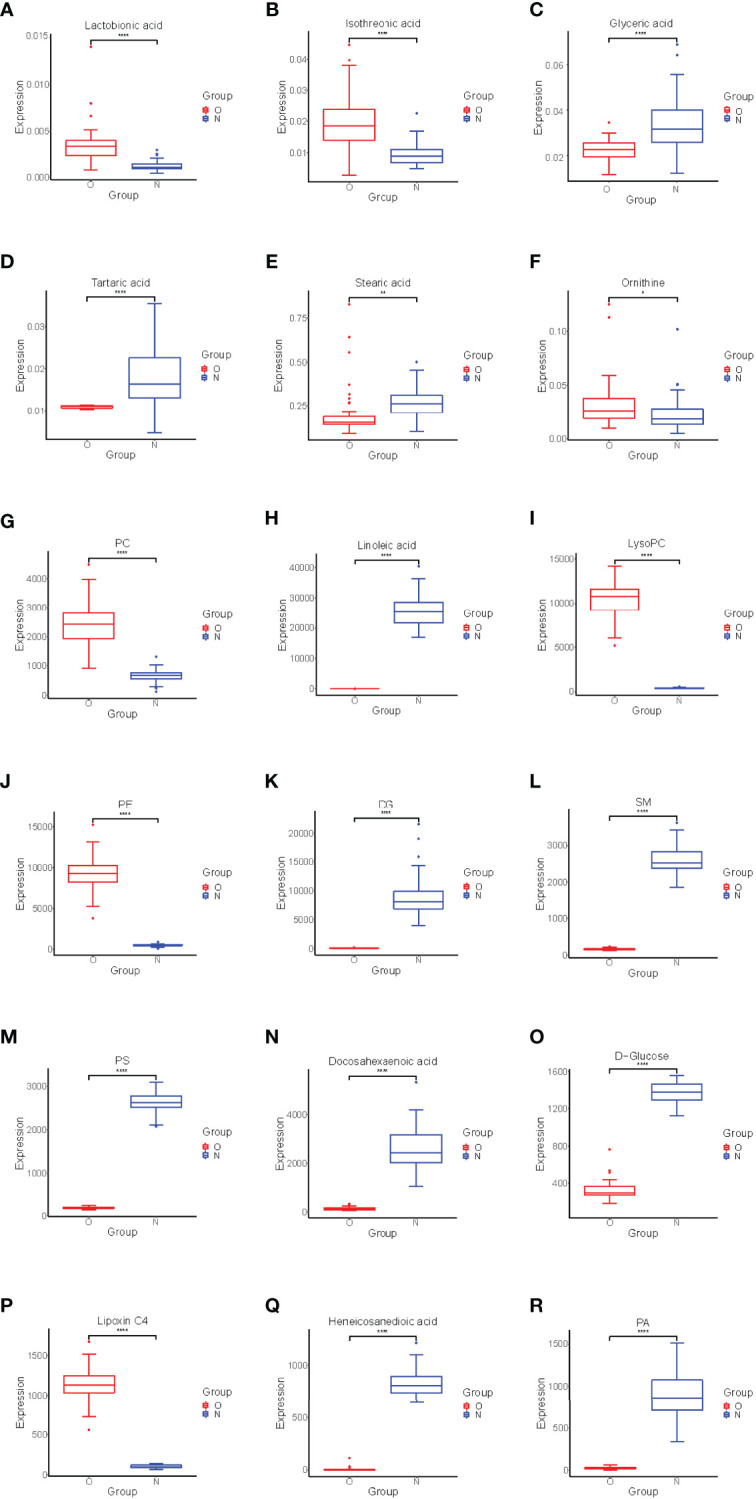
Box-and-whisker plots showing the relative levels of selected potential biomarkers for the postmenopausal women with OP. **(A–F)** were found by GC-MS, **(G–R)** were found by LC-MS. The red box on the left represents the case group, and the blue box on the right represents the healthy control group. Horizontal line in the middle portion of the box, median; bottom and top boundaries of boxes, lower and upper quartiles; whiskers, 5th and 95th percentiles. *p < 0.05, **p < 0.01, ***p < 0.001, ****p < 0.0001. PC, Phosphatidylcholine; LysoPC, lysophosphatidylcholine; PE, phosphatidylethanolamine; DG, Diacylglycerol; PS, phosphatidylserine; SM, sphingomyelin; PA, phosphatidic acid.

**Figure 4:**
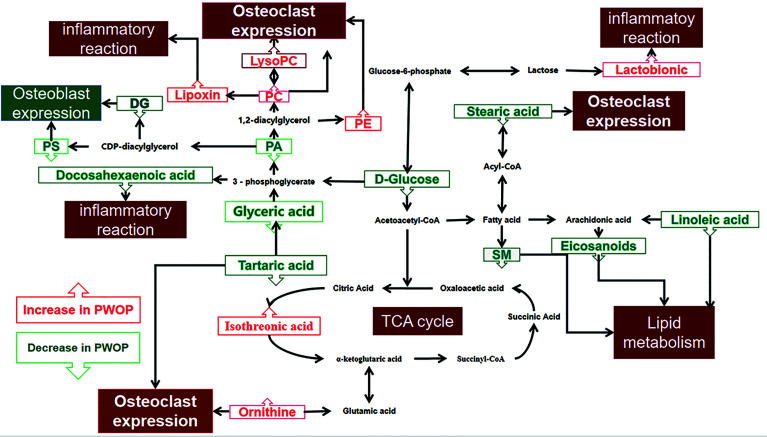
Altered metabolic pathways for the most relevant distinguishing metabolites (potential biomarkers) between the case group and healthy control group. The metabolites with red border were upregulated in the case group, whereas those with green border indicate metabolites that were downregulated. PC, phosphatidylcholine; LysoPC, lysophosphatidylcholine; PE, phosphatidylethanolamine; DG, diacylglycerol; PS, phosphatidylserine; SM, sphingomyelin; PA, phosphatidic acid.

Significant differences between groups can also be shown by potential biomarker box-and-whisker plots (**Figure 3**). By database searching (KEGG) and consulting relevant literature, we found that these metabolites are mostly related to glucose, amino acids, and choline metabolism and also have some relationship with inflammatory response. These metabolic pathways often have a close relation to the changes in the marrow microenvironment in the bone marrow, which can eventually lead to the changes in osteoclast differentiation and oxidative stress. As shown in **Figure 4**, we can more intuitively reflect the relationship between these metabolites by drawing the metabolic pathway map of these metabolic markers with significant differences.

## Discussion

Metabonomics is widely regarded as the most phenotypic omics by identifying and quantifying small molecular metabolites (27). Because of its inherent sensitivity, metabonomics is the most powerful method to study local and specific stimulus responses and pathogenesis. It can detect subtle changes in biological pathways to obtain clear biochemical information about disease mechanisms, to help us understand the process of various physiological conditions and abnormal processes (28). OP is a metabolic disease that eventually causes the continuous decrease in bone mass and the deterioration of the bone microstructure. Metabonomics analysis can further examine the pathological process of OP and identify the reaction to drugs in each stage of OP treatment (29). Untargeted GC-MS combined with LC-MS was used for the first time to describe the metabolism of 50 Chinese postmenopausal women with OP and 50 Chinese postmenopausal women with normal BMD for our study. Through multivariate analysis, we found that postmenopausal women with OP/normal bone mass had a large number of metabolites with significant differences. This shows that the PCA and PLS-DA/OPLS-DA models established by using normal serum metabolites of Chinese postmenopausal women with OP and Chinese postmenopausal women with normal bone mass have high sensitivity and specificity.

In previous metabonomics studies, some potential biomarkers of osteoporosis have been found. These metabolites are mainly concentrated in fats [e.g., phosphatidylinositol, phosphatidic acid, sphingolipid (15), linoleic acid, oleic acid, arachidonic acid, and 11, 14-eicosadienoic acid (17)] and amino acids [e.g., glutamine (14), 4-aminobutyric acid, proline, aminopropionitrile, threonine, methionine (15), leucine, isoleucine, and taurine (17)]. Our study also found some new potential biomarkers, which greatly enriched the database of potential markers of osteoporosis and provided more directions for the diagnosis of osteoporosis. At the same time, we also found that some potential biomarkers we detected this time were also found in previous experiments, which further shows that metabonomics has a certain repeatability in the study of osteoporosis. It also shows that these repeatedly verified potential biomarkers have greater potential to become markers for the diagnosis of osteoporosis.

Adult bone is a multifunctional organ that is constantly reconstructed. In adults who have normal bone mass, the resorption of osteoclast bone and the formation of osteoblast bone bones have a delicate balance. When this balance is broken, it leads to OP and other bone diseases (30). Before menopause, estrogen can reduce oxidative stress in bone and bone marrow, so as to maintain the balance of the bone microenvironment and keep bone strength in the normal range (31). However, this balance is slowly broken after menopause. In addition, the expression of the receptor activator of nuclear factor-b ligand (RANKL) will be overexpressed with estrogen deficiency, which is also a reason for the increase in bone resorption to achieve OP (32). Bone resorption of osteoclasts consumes a large amount of energy, which makes glycolysis and oxidative phosphorylation to speed up. Patients with OP usually have fatty acid disorder and abnormal amino acid metabolism, which have promoted the occurrence and development of OP (33).

With the enhancement of energy metabolism, the citric acid cycle is also enhanced. As an important intermediate product of the citric acid cycle, the concentration of isotricarboxylic acid in serum is also increased (34), a large amount of glucose is used, and the glucose concentration becomes lower. The above mechanism may explain that the serum glucose concentration is low and the isothreonic acid concentration is high. Lactonic acid has strong antioxidant capacity, can chelate Fe^3+^, and can reduce the tissue damage caused by hydroxyl free radicals produced by ion catalysis (35). Therefore, the increase in lactonic acid may be related to the reduction in tissue damage caused by hydrogen and oxygen free radicals produced by the enhancement of energy metabolism.

Fat and bone have a very complex relationship with each other, and this correlation is widely reflected in both systematic and local aspects. Local effect is mainly reflected in the change of the bone marrow microenvironment and the expression of fat with other bone cells (36). *In vitro*, under the pro-inflammatory stimulation of TNF-α and IFN-γ, bone marrow mesenchymal stem cells (MSCs) were activated and the metabolism of PE, PS, and lysoPC was affected (37). The content of PE increased during osteoclast differentiation (38), and LysoPC can be transformed into phosphatidylcholine (PC). LysoPC can promote osteoclast differentiation and increase intracellular free calcium concentration (39). Our results also support the positive correlation between PE, lysoPC, and PC and decreased BMD.

Some *in vitro* experiments show that high levels of PS can stimulate osteoblasts and promote the deposition of mineral substances in bone tissue (40). Diacylglycerol (DG), released from membrane lipids, is a cellular mediator that was critical for the regulation of inflammation and disease (41), which can promote protein kinase C (PKC) expression. PKC activates calcium absorption and increases the cAMP concentration in osteoblasts (42). Phosphatidic acid (PA) is the main metabolite in the synthesis of DG, so the decrease of DG, PA, and PS may be related to the inhibition of osteoblast activation in patients with OP, which leads to the further development of OP. In Diana Cabrera’s report (15), there are also results consistent with ours.

In other studies, stearic acid (43) and tartaric acid (44) have obvious inhibitory effects on osteoclasts. The final metabolite of tartaric acid is glyceric acid. At the same time, glyceric acid can further isomerize into sugar or further participate in glycolysis to meet the energy metabolism of osteoclasts (45). The decrease in stearic acid, tartaric acid, and glyceric acid may reduce the inhibitory effect of fat on osteoclasts, which will be accompanied by the decrease in human bone mass and eventually develop into OP. The findings suggest that low levels of stearic acid, tartaric acid, and glyceric acid in serum may predict low BMD.

In the mouse model of OP, lipoxygenase (LOX) gene expression leads to an increase in the concentration of lipoxin, which can produce endogenous anti-inflammatory effects, which is related to the decrease in bone strength in the mouse model of OP. Some eicosanoids are related to allergic reaction and inflammation and play a pro-inflammatory role, which is opposite to lipoxin (46). This helps to explain the results that we detected an increase in lipoxin C4 and a decrease in docosahexaenoic acid in the patient group. In previous studies, it was found that long-term OP would reduce the levels of arachidonic acid, docosahexaenoic acid, and sphingomyelin (SM) (47). Arachidonic acid is formed by linoleic acid metabolism *in vivo*, which is consistent with the change trend. Therefore, in our study, we found that the levels of docosahexaenoic acid, sphingomyelin, and linoleic acid in the experimental group were at a relatively low level. In the study of the macrophage signaling pathway, it was found that the animal model had OP, which showed that the expression of hyperactive osteoclasts increased. These macrophages produced *in vivo* have high arginase levels and produce ornithine (48). This is also similar to our test results, which confirmed that the ornithine concentration of OP patients is higher.

Finally, through the correlation analysis of 18 metabolites screened and 2 bone turnover markers (TRACP5b, BAP) in this study, it is found that only 3 metabolites are significantly correlated with them, while other metabolites have only a non-significant correlation. Firstly, bone turnover markers are not the gold standard for diagnosis of osteoporosis. Bone turnover markers are more effective in determining the response to osteoporosis treatment and as a reference in the diagnosis of secondary osteoporosis, but the prediction effect on primary osteoporosis is not very good. Secondly, there are day-to-day changes in the concentration of bone turnover markers (49), which may be the reason why we get such results.

Overall, the metabolomic profiles we obtained were promising. These potential biomarkers have great biological significance for the diagnosis and recurrence monitoring of postmenopausal OP women. However, we admit that our study is not enough. First, the number of samples is not rich enough, and the samples are mostly from Sichuan Province and surrounding areas; the result should be verified in more postmenopausal OP women in the future. Secondly, this study only uses serum as the sample for exploration, and the results are relatively incomplete. Therefore, more kinds of samples can be selected for OP metabolome research in the future, such as bone marrow and urine, so as to establish a more complete metabolic database. In addition, there is a lack of absolute qualitative and quantitative data of substances that untargeted metabonomics may produce a lot of false positive signals. The potential biomarkers could be studied by targeted metabonomics in the next step. Finally, the results of this study are only for postmenopausal women and can be further explored in OP male patients.

## Conclusions

The metabolism analysis of postmenopausal women with OP is the first time to study by untargeted GC/LC-MS on serum to obtain more comprehensive metabolomic characteristics and screen out a large number of potential biomarkers with significant differences. Through multivariate data analysis and metabolic pathway analysis, most of these metabolic markers are related to the disorder of glucose metabolism, amino acid metabolism, and lipid metabolism and influence the bone microenvironment and the homeostasis changes of the whole body in OP women. 18 metabolites with significant differences were screened, which is important in these metabolic pathways,which are judged to have great potential as potential biomarkers of OP in postmenopausal women. In a further study, we need to conduct more validation experiments to prove that these biomarkers we found can be widely used in clinical work.

## Data Availability Statement

The raw data supporting the conclusions of this article will be made available by the authors, without undue reservation.

## Ethics Statement

The studies involving human participants were reviewed and approved by the General Hospital of Western Theater Command. The patients/participants provided their written informed consent to participate in this study.

## Author Contributions

Conceptualization: JK, CH, LC, ZZ, WW, LT, and DL. Carried out the experiments: JK, WZ, WG, and NX. Analyzed and interpreted data: JK, CH, LC, ZZ, LT, WZ, WG, and NX. Writing—original draft: JK, CH, LC, and ZZ. Review and editing: WW and DL. Funding acquisition: WW. All authors contributed to the article and approved the submitted version.

## Funding

The present work was supported by the Department of Science and Technology of Sichuan Province (CN) Project (grant agreement 2019YJ0278) and the General Hospital of Western Theater Command Project (grant agreement 2021-XZYG-B05).

## Conflict of Interest

The authors declare that the research was conducted in the absence of any commercial or financial relationships that could be construed as a potential conflict of interest.

## Publisher’s Note

All claims expressed in this article are solely those of the authors and do not necessarily represent those of their affiliated organizations, or those of the publisher, the editors and the reviewers. Any product that may be evaluated in this article, or claim that may be made by its manufacturer, is not guaranteed or endorsed by the publisher.
